# Anatomical structures and needling method of the back-shu points BL18, BL20, and BL22 related to gastrointestinal organs: A PRISMA-compliant systematic review of acupoints and exploratory mechanism analysis

**DOI:** 10.1097/MD.0000000000029878

**Published:** 2022-10-28

**Authors:** Yeonwoo Cho, Yaejin Han, Yeji Kim, Sihyun Han, Kichang Oh, Hyocheong Chae, Chu Hongmin, Myungseok Ryu

**Affiliations:** a College of Korean Medicine, Dongguk University, Ilsan City, Republic of Korea; b Academic Affairs Board, Korean Medical Society of Soft Tissue, Seoul, Republic of Korea; c Daecheong Island Branch Office of a Ongjin Public Health Center, Incheon, Republic of Korea; d Daemyung Korean Medicine Clinic, Seoul, Republic of Korea.

**Keywords:** acupuncture, back-shu points, BL18, BL20, BL22, systematic review

## Abstract

Acupuncture treatment on back-shu points (BSPs) has received attention owing its ability to control the function of visceral organs. We aimed to conduct a systematic review to provide detailed information on the effectiveness and safety of BL18, BL20, and BL22 on the digestive system in terms of soft tissue and anatomical structure and assist in the appropriate application.

**Methods:**

Medline, Cochrane Library, EMBASE, OASIS, RISS, and CNKI were searched from their inception to July 2021. This systematic review included randomized controlled trials, controlled clinical trials, case series, and case reports that addressed anatomical structures or needling methods of BL18, BL20, and BL22.

**Results:**

In total, 115 articles were included from the 7 electronic databases. One hundred eight articles described the depth and method. A total of 96 articles described depth, 86 articles described the angle, and 74 articles described both. Seventy-nine articles described the target muscles and anatomical structure. Acupuncture on BSP is effective in gastrointestinal diseases because of compression of the spinal nerve, sympathetic nerve hyperactivity, and connection of the diaphragm. By reviewing each study’s acupuncture method and target muscles, we analyzed the angle and depth of the needle that effectively leads to therapeutic response.

**Conclusions:**

This study provides guidance on applying needles in terms of anatomical structures to yield therapeutic responses. However, few studies have assessed how to effectively stimulate BSP to trigger digestive effects and their mechanisms. Additional studies on the relationship between BSP and the digestive system are needed to use these acupoints for digestive diseases.

## 1. Introduction

Acupuncture at certain points on the body has been shown to have analgesic and therapeutic effects in various diseases.^[1]^ The traditional East Asian medical textbooks provide many instructions regarding selecting the acupoints according to the symptoms or diseases.^[2,3]^ For selecting accurate acupoints, various theories were oriented to traditional East Asian medicine theory. Many attempts have been made to determine the physiological relationships between acupuncture stimulations and therapeutic responses.^[4]^

As each acupoint has its therapeutic effects and physiological functions, back-shu points are known for treating visceral diseases.^[5]^ Back-shu points are acupoints located parallel to the vertebra and located on the urinary bladder meridian. In addition, each point is located on approximately the same horizontal plane as that of the related organs. For example, the back-shu point of the kidney is on the same level as the back as the kidney. Several studies have selected back-shu points for treating various visceral dysfunctions or diseases such as palpitation, urinary bladder contractions, stomach and intestinal mobility dysfunctions, and pancreatic secretions.^[6–8]^

However, the general knowledge of accurate needling or mechanisms of the back-shu points is insufficient. Therefore, we systemically reviewed studies on back-shu points and selected 3 back-shu points related to gastrointestinal (GI) functions. The 3 acupoints were BL18 (liver-shu point), BL20 (spleen-shu point), and BL22 (triple heater-shu point), which are related to the intestines, especially digestive organs. According to Hwang’s research, those 3 acupoints are related to anatomically T9 spine line. BL 22 is on the dermatome, which is innervated by T9 spinal neuron. And BL18 and BL20 are related to visceral organs in which T9 spinal neuron is innervated.^[9]^ So, we expect that this review of the anatomical structures and needling methods of those 3 back-shu points related to digestive organs will help ensure their appropriate application.

## 2. Methods

**Figure 1.**
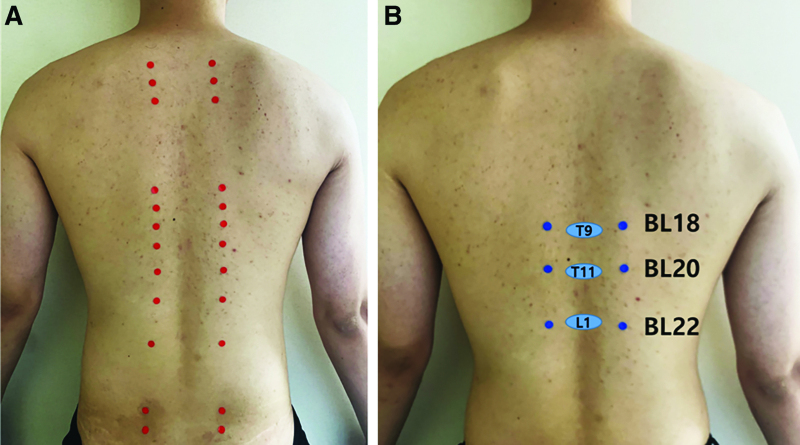
Locations of back-shu points. (A) Total back-shu point`s locations. (B) Locations of BL18, BL20, BL22. L1 = 1st lumbar vertebra bone, T9; 9th thoracic vertebra bone, T11 = 11th thoracic vertebra bone.

The purpose of this study was to determine the anatomical structure and needling depth of back-shu points related to GI diseases such as BL18, BL20, and BL22. Locations of back-shu points and BL18, BL20, and BL22 are shown in Figure 1.

### 2.1. Classical medical book research

According to traditional medicine classics in Korea, such as Donguibogam (東醫寶鑑), Chimgugapeulgyeong (針灸甲乙經), and Chimgudaeseong (針灸大成), acupoints BL18, BL20, and BL22 have therapeutic effects on intestinal diseases such as gastritis and intestinal bowel disease.^[10]^ This implies that these acupoints affect digestive systems, and each of them may be named according to the related organs (Table 1).

**Table 1:** Relationships between main effect of back-shu points and spinal cord.

Acupoint	Spinal cord	Therapeutic effects on the GI disease
BL18	T9	Hepatitis (肝炎), cholecystitis (膽囊炎), jaundice (黃疸), bitter taste (口苦), dryness of the tongue (舌乾), indigestion (消化障碍), gastritis (胃炎), constipation (便秘), vomiting (吐食), headache (頭痛)
BL20	T11	Dyspepsia (消化不良), gastroenteritis (胃腸炎), gastritis (胃炎), gastric ulcer (胃潰瘍), gastric ptosis (胃下垂), spleen and stomach qi deficiency (脾胃虛弱), fullness of hypochondrium/feeling fullness at the flank (脇下滿), abdominal distension (腹脹), ascites (腹水), edema (浮腫), gastralgia (胃痛), jaundice (黃疸), diabetes mellitus (糖尿病), bleeding disorders (出血性疾病), quadriplegia (四肢不收), chronic indigestion (積聚), diarrhea (泄痢), abdominal pain (腹痛), severe emaciation (多食羸瘦)
BL22	L1	Gastrospasm/proctospasm (腸痙攣), gastritis (胃炎), vomiting (嘔吐, 吐逆), abdominal distension (腹脹), rugitus (腹鳴), dyspepsia (消化不良), gastroenteritis (胃腸炎), nocturia (夜尿症), sexual impotence (陰萎), ascites (腹水), dysuria (小便不利), diarrhea (泄痢), dysentery (痢疾), stiffness and pain in the lumbar and back (腰脊强痛)

### 2.2. Search strategy

This systematic review was registered in the International Prospective Register of Systematic Reviews (PROSPERO database at https://www.crd.york.ac.uk/PROSPERO) CRD42021272840 and followed the guidelines provided by the Preferred Reporting Items for Systematic Review and Meta-Analysis statement.^[11]^ Because this study will quantitatively synthesize data from previously published papers, institutional review board approval and consent of the subject are not required.

#### 2.2.1. Data source.

The following 6 databases were searched from their inception to July 2021: Medline (Pubmed, https://pubmed.ncbi.nlm.nih.gov/), Cochrane Library (CENTRAL, https://www.cochranelibrary.com/), EMBASE (https://www.embase.com), OASIS (https://oasis.kiom.re.kr/), RISS (https://www.riss.kr/), and China National Knowledge Infrastructure(https://oversea.cnki.net/index/). In this review, we included randomized controlled trials, controlled clinical trials, case series, and case reports addressing the anatomical structure or needling methods of BL18, BL20, and BL22.

#### 2.2.2. Study selection.

The selection process was conducted independently by 2 authors using referencing software. In the primary study, they reviewed the titles and abstracts and selected suitable studies. Next, the full texts of the selected studies were reviewed and confirmed inclusion. Studies on manual acupuncture were included, and studies that did not mention the anatomical structures or needling depth and applied other interventions such as moxibustion and scraping therapy were excluded. The search strategies for each database are shown in Table 2.

**Table 2:** Searching strategy

MEDLINE(Pubmed)
1. “Acupuncture”[MeSH Terms]
2. “Acupuncture Points “[MeSH Terms]
3. “Acupoint”[Title/Abstract]
4. “Back-shu Point*”[Title/Abstract]
5. “Backshu*”[Title/Abstract]
6. “Ganshu”[Title/Abstract]
7. “BL18”[Title/Abstract]
8. “Pishu”[Title/Abstract]
9. “BL20”[Title/Abstract]
10. “Shanjiaoshu”[Title/Abstract]
11. “BL22”[Title/Abstract]
12. #1 OR #2 OR #3 OR #4 OR #5 OR #6 OR #7 OR #8 OR #9 OR #10 OR #11
13. “Anatomy”[MeSH Terms]
14. “Safe Depth”[All Field]
15. “Needling Depth”[All Field]
16. “Depth”[Title/Abstract]
17. “Angle”[Title/Abstract]
18. #13 or #14 or #15 or #16 or #17
19. #12 and #18
Filters applied: Case Reports, Clinical Trial, Meta-Analysis, Observational Study, Pragmatic Clinical Trial, Randomized Controlled Trial, Review, Systematic Review, Humans
EMBASE
#1 “Anatomy”/exp OR “Safe Depth” OR “Needling Depth” OR “ Depth” OR “Angle”:ab,ti
#2 (“acupuncture”/exp OR “acupoint*”/exp OR “ Back-shu Point”/exp OR “Backshu*”/exp OR Backshu OR BL18$ OR Ganshu OR Pishu OR Shanjiaoshu OR BL20$ OR BL22$)
#3 #1 AND #2 AND ([cochrane review]/lim OR [systematic review]/lim OR [meta analysis]/lim OR ([clinical trial]/lim OR ([observational study]/lim)
Cochrane
#1 Acupuncture [Mesh]
#2 Acupuncture Points
#3 Acupoint
#4 Back-shu Point
#5 Backshu*
#6 Ganshu
#7 BL18
#8 Pishu
#9 BL20
#10 Shanjiaoshu
#11 BL22
#12 #1 OR #2 OR #3 OR #4 OR #5 OR #6 OR #7 OR #8 OR #9 OR #10 OR #11
#13 Anatomy[explode all tree] [Mesh]
#14 Safe depth
#15 Needling depth
#16 Depth
#17 Angle
#18 #13 OR #14 OR #15 OR #16 OR #17
#19 #11 AND #18
*Filter: in Cochrane review
CNKI
(TI, SU = “背俞” OR TI, SU = “背腧” OR TI, SU = “肝俞” OR TI, SU = “脾兪” OR TI, SU = “三焦俞” OR SU = “Back shu”) AND (TI, SU, AB = “解剖” OR TI, SU = “分析” OR TI, SU = “针刺”OR TI, SU = “深度” OR TI,SU = “深浅”)
Korean Database (RISS, OASIS, DBPIA)
“간수혈” OR “비수혈” OR “삼초수혈” OR “배수혈” OR “간수” OR “비수” OR “삼초수”

#### 2.2.3. Inclusion criteria.

The inclusion criteria were as follows: studies related to anatomical structure, studies related to needling depth or angle, and clinical studies such as prospective randomized controlled trials, observational studies, and case reports.

#### 2.2.4. Exclusion criteria.

The exclusion criteria were as follows: out-of-scope studies not addressing BL18, BL20, and BL22; posters; studies without abstracts; animal studies; studies for which the full text was not available; studies not written in English, Chinese, or Korean; and studies that were not peer-reviewed.

#### 2.2.5. Data extraction.

More than 2459 articles were retrieved and reviewed by 2 independent researchers. Our focus was on full articles describing the needling methods or anatomical structures. However, we also reviewed articles that provided brief descriptions of the clinical studies that mentioned detailed acupuncture needling methods. Based on the inclusion and exclusion criteria, 115 studies were included in this review after removing duplications and irrelevant studies.

## 3. Results

### 3.1. Description of the included studies

**Figure 2.**
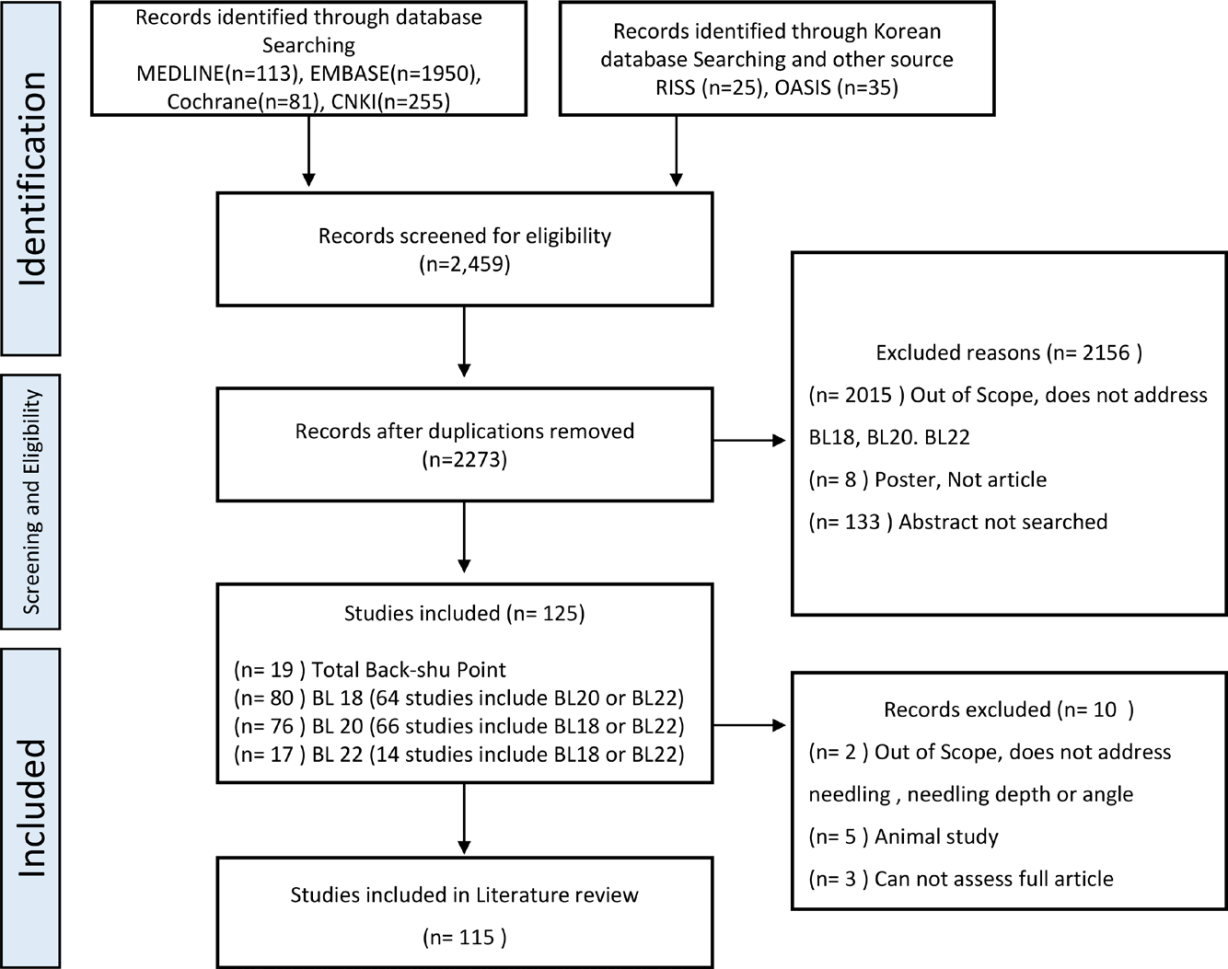
Flowchart of this review.

**Figure 3.**
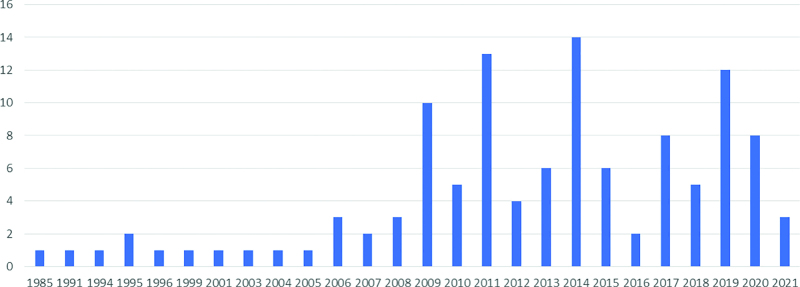
Publication year and the number of articles.

A total of 115 articles were included from the 7 electronic databases. The selection process and reasons for exclusion are shown in Figure 2. In total, 112 articles were written in Chinese and 3 were written in English. According to the analysis of the countries in which the study was conducted, 114 were clinical trials conducted in China, and 1 was conducted in Japan. The articles were published between 1985 and 2021. The publication years and number of articles are shown in Figure 3.

### 3.2. Needling depth and angle of BL18, BL20, and BL22

Among the 115 articles, the depth and method were described in 108 articles. A total of 96 articles described the depth, 86 described the angle, and 74 described both. The overall results of included studies are displayed in Supplement 1 (Supplemental Digital Content, http://links.lww.com/MD/G993). Feng^[12]^ suggested 15 to 20 mm depth when acupuncturing 90 degrees, and Shi et al^[13]^ suggested 9 to 16 mm depth. The needling depths of included articles are shown in Table 3.

**Table 3:** Needling depths and angles extracted from the searched articles.

	BL18		BL20		BL22		Total back-shu		Wu-Zang back-shu	
	Depth	N	Depth	N	Depth	N	Depth	N	Depth	N
Average (not mention angles)	20.91	68	22	67	22.45	15	19.7	9	19.94	7
Average (30°)	25.05	2	27.3	2	–	–	–	–	–	–
Average (45°)	18.89	14	28.19	10	16.77	4	17.27	3	15	2
Average (60°)	29.8	2	15.75	2	32.1	1	–	–	–	–
Average (75°)	37.45	2	13.91	2	–	–	–	–	–	–
Average (90°)	18.79	13	22.74	16	18.33	3	23.01	2	21.53	3
Average (only mention oblique needling method)	24.87	17	21.37	18	27.62	5	–	–	22.5	2
Do not mention both angle and depth	–	30	–	23	–	4	–	8	–	0

### 3.3. Featured research of needling insertion on BL18, BL20, and BL22

Several studies have described the detailed needling depth or angles. Li used a fixed cadaver and measured the depth using a surgical microscope. In this study, BL18 was related to the trapezius and middle accessory neurovascular bundles. BL18, BL20, and BL22 were related to the latissimus dorsi and canal nerves (axillary arteries and veins).^[14]^ In Zhao’s study, different needling depths were set depending on the disease. The BL18 reference depth was 3.03 cm (1 cun) and that for hypertension was 1.54 to 2.62 cm (0.5–0.8 cun). The BL20 reference depth for diabetes was 4.57 to 6.06 cm (1.5–2 cun). The reference depth for constipation was 1.54 to 2.62 cm (0.5–0.8 cun).^[15]^ Feng divided the subjects into 2 groups according to obesity. This study included patients with poststroke depression; researchers applied a reference depth of 3.03 cm (1 cun) to subjects with obesity and that of 1.54 cm (0.5 cun) to subjects without obesity.^[12]^ Cong et al^[16]^ presented a safe needling depth of BL18. According to Cong’s research, safe needling depth categories are gender, right and left, and angles. If needling insertion was performed vertically, the dangerous needling depth was 33.47 ± 0.87 mm in males and 30.02 ± 1.31 mm in the females on the left BL18 and 35.32 ± 0.77 mm in males and 34.45 ± 0.85 mm in female on the right BL18. When needling insertion was tilted, the dangerous needling depth was 48.55 ± 1.44 mm in males and 47.83 ± 1.29 mm in females on the left BL18 and 50.44 ± 1.60 mm in males and 49.30 ± 1.30 mm on the right BL18.^[16]^

Shi et al used computed tomography to measure the needling depth of the back-shu point. In this study, the depth of BL18 was 11.4 ± 4.7 mm (left) and 11.2 ± 5.0 mm (right) in males and 10.3 ± 4.6 (left) and 10.1 ± 5.1 (right) in females. The depth of BL20 was 10.9 ± 5.6 (left) and 10.3 ± 5.3 (right) in males and 9.8 ± 5.5 (left) and 10.2 ± 5.1 (right) in females.^[13]^

### 3.4. Anatomical structures mentioned in previous research

Seventy-nine articles mentioned the anatomical target of acupoint needling as muscles. In Li’s^[14]^ study, BL18 targeted stimulation of the trapezius muscle, the middle accessory neurovascular bundle, the latissimus dorsi, and latissimus dorsi canal nerves. BL20 and BL22 targeted the stimulating and latissimus dorsi, latissimus dorsi canal nerves, and soft tissue around the axillary arteries and veins. He’s study mentioned that when needling on the back-shu point is performed, the needle should be inserted deep enough to reach the transverse spinal process.^[17]^ Dong et al^[18]^ mentioned needling on the BL18 and BL20; the physicians targeted the muscular lesion side’s tendon. The study by Li^[19]^ was conducted on patients with chronic urticaria; they performed needling on the BL18-targeted cutaneous branches of the posterior ramus of the 9th and 10th thoracic nerves and related arteries and veins of soft tissue.

### 3.5. Average needling depth and angle

The average depth of BL18, BL20, and BL22 acupoints was 20 to 22 mm in vertical insertion and 24 to 27 mm in oblique insertion.

## 4. Discussion and conclusions

### 4.1. Exploratory mechanism of therapeutic effects on GI disease

We aimed to explore why these acupoints act on GI organs. There are many structures around the facet joint of the spinal bone, including connective tissue such as the intertransverse ligament and muscles such as the multifidus, longissimus thoracis, and spinalis thoracic.^[20]^ Inflammation of the intertransverse ligament or increased tension of muscles can narrow the intervertebral foramen, leading to compression of the spinal nerve trunk. Acupuncture in these regions can release tension and decrease inflammation.

#### 4.1.1. Resolving overexcitabilities and ischemic status of the sympathetic nerve due to pressure.

Acupuncture can resolve the overexcitabilities and ischemic status of the sympathetic nerve due to pressure. Focal compression of the nerve inhibits the Na+/K+ ATPase pump and causes axonal depolarization^[21–23]^ If the spinal nerve trunk is compressed, the vasa nervorum of sympathetic preganglionic fibers is pressed, leading to focal ischemia. Focal ischemia can decrease adenosine triphosphate production, and the Na+/K+ ATPase pump can fail to maintain homeostasis, leading to an increase in extracellular K+ concentration. Therefore, focal compression can lead to membrane depolarization.^[24]^ As a result, the resting membrane potential increases, and the threshold of nerve cells is reduced, causing membrane hyperexcitability. Hyperexcited sympathetic preganglionic fibers can be excited easily and produce abnormal excitation signals.^[25,26]^ Acupuncture needling on the thoracic spinal muscle can release this ischemia status.

#### 4.1.2. Stimulating the nerve pathway of sympathetic preganglionic fibers.

The axons of neurons synapse on postganglionic neurons are located in the paravertebral and prevertebral ganglia. Sympathetic preganglionic fibers from T9 and T11 reach the celiac ganglion and superior mesenteric ganglion, and postganglionic fibers innervate the stomach, small intestine, and ascending colon. Sympathetic preganglionic fibers from L1 reach the inferior mesenteric ganglion and postganglionic fibers supplying the descending colon, rectum, and bladder.^[27]^

The sympathetic nerve prominently innervates the enteric ganglia, GI arterioles, and circular muscle of the sphincters. Few sympathetic fibers innervate muscle layers of the nonsphincter; lymphoid tissue; and mucosa of the stomach, intestines, gallbladder, and immune cells. Postganglionic nerves, whose cell bodies are in the paravertebral ganglia and supply the GI tract directly, help vasoconstrictor neurons control the blood vessels. Vasoconstrictor neurons influence motility and sympathetic neurons that innervate the secretomotor neurons. These innervations enable sympathetic nerves to participate in GI diseases. Sympathetic neurons inhibit intestinal motility, and activation of sympathetic neurons during the GI inhibitory reflex inhibits the activation of enteric neurons. In these processes, the GI tract’s actions normalize the transition of intestinal content.^[28–30]^ Water and electrolyte transport and the change in blood flow to the organs are also influenced by gastric functions. The sympathetic nerve controls secretomotor neurons to maintain fluid balance by controlling water and electrolyte transport. Sympathetic nerves, which receive abnormal excitation signals, can increase the inhibition of fluid secretion into the lumen of the intestine, thereby disturbing the balance of fluid exchange in the GI tract. Sympathetic neurons also strongly innervate the arteries of the GI tract, which carry blood from the heart and veins, such as the mesenteric veins and portal veins, which carry blood from the gut to the liver. The axon of the hyperexcited sympathetic preganglionic nerve produces more norepinephrine, and norepinephrine works on α1 receptors. It activates smooth muscles of visceral arteries, thereby reducing blood flow to the GI tract. Sympathetic dysfunction can result in inflammatory bowel diseases such as ulcerative colitis and Crohn disease.^[31]^ Further, GI dysfunctions may occur due to impairment of sympathetic nerves or pathways owing to accumulation of old lesions or imbalances. Acupuncture stimulation of the nerve pathway can solve this problem.

Also, there is a case report on treating GI tract diseases such as refractory laryngopharyngeal reflux by targeting the release of multifidus hyperexcitability of T5 and T6 sympathetic preganglionic fibers.^[32]^ Sympathetic preganglionic fibers from T5 and T6 travel via the greater thoracic splanchnic nerves that reach the celiac ganglion, and the postganglionic fibers innervate the lower esophageal sphincter and vascular smooth muscles. By reducing intervertebral pressure, nerve compression on T5 and T6 is released, which reduces lower esophageal sphincter relaxation and improves blood flow to the mucosa. Focal acupuncture on BL18, BL20, and BL22 can reduce inflammation of the intertransverse ligament or tension of the spinal muscles, thereby improving pathological conditions of the sympathetic nervous system that strongly innervates the GI tract and affects motility, secretion, blood flow, and immune cells.^[33]^

#### 4.1.3. Stimulating soft tissue around diaphragm and GI organs.

The diaphragm, which separates the thorax from the abdomen, is the primary ventilation muscle. The anterior and lateral parts of the diaphragm are attached to the lower 6 ribs, costal cartilage, inferior sternum, and xiphoid process. The posterior part of the diaphragm is attached to the median, medial, and lateral arcuate ligaments.

The diaphragmatic crus attaches the diaphragm to the upper lumbar vertebral bodies and disks. The median arcuate ligament joins these cruses across the aorta and the celiac artery. Hypertrophy or low position of the median arcuate ligament can compress the celiac artery, called median arcuate ligament syndrome, which is characterized by foregut ischemia, including epigastric pain, nausea, vomiting, and weight loss.^[34,35]^

The medial arcuate ligaments attach the diaphragm to the L1 or L2 vertebral body and the transverse processes of L1. The lateral arcuate ligaments extend from the transverse processes of T12 to the mid-portion of the 12th rib, which enables a posterior approach to the diaphragm with acupuncture. However, several acupuncture points are related to the digestive system, including BL18, BL20, BL21, and BL22, BL20, BL21, and BL22 are on the back, 1.5 cun lateral to the lower border of the spinous process of the T11. Acupuncture can attach the lateral arcuate ligaments to the superior region of the 12th rib if it is acupunctured obliquely on BL20 in the lower lateral direction. BL21 is on the middle back, 1.5 cun lateral to the lower border of the spinous process of the T12. Acupuncture can be performed on the L1 transverse process where the median arcuate ligament and lateral arcuate attach if it is acupunctured obliquely on BL21 in the lower medial direction. In addition, acupuncture can attach the lateral arcuate ligaments to the inferior region of the 12th rib if it is acupunctured obliquely on BL21 in the upper lateral direction.

The posterior region of the diaphragm is much lower than the anterior region; therefore, the diaphragm is connected to the superior part of the dorsal mesentery and ventral mesentery. These 2 mesenteries are attached to the liver, stomach, and spleen, and each organ is in the right position. The stomach is connected to the diaphragm via the gastrophrenic ligament. The lesser omentum and hepatogastric ligament also connect the liver and stomach.^[20]^

The esophageal hiatus is at the T10 level and contains the esophagus, vagus nerve, and sympathetic nerve branches. Acupuncture on the diaphragm can stimulate the vagus nerve and sympathetic nerves, thereby regulating digestive function directly. In addition, the diaphragm exerts external pressure at the esophageal hiatus to prevent gastroesophageal reflux, suggesting a treatment point for gastroesophageal disease due to diaphragm dysfunction.^[34]^

Considering attachment of the diaphragm to the thoracic, lumbar vertebra, and 12th rib, acupuncture on the BL20 or near back-shu acupoints may affect GI function directly through the connection between the diaphragm and the GI tract or esophageal hiatus and indirectly by structures connected with the diaphragm, including the lesser omentum and hepatogastric ligament.

### 4.2. Limitation

The present study aimed to explore the needling depth and angle of the back-shu points related to GI disease. This systematic review presented accurate acupuncture needle insertion guidelines for BL18, BL20, and BL22. We analyzed 115 articles in this review, and the results showed that the average depth of these acupoints was 20 to 22 mm in the vertical insertion and 24 to 27 mm in the oblique insertion.

Although many classic medical books suggest applying these back-shu points to digestive diseases, follow-up research on how these acupoints have therapeutic effects on GI disease is insufficient. Standardization of needling or the target of the acupuncture action for acquiring the therapeutic effects on the GI disease is also not presented precisely.

Therefore, we selected 3 acupoints of back-shu points, BL18, BL20, and BL22, which are known to treat digestive disorders. BL18 and BL20 feature the liver and pancreas at the back-shu points. In East Asian medicine, liver and pancreas play the main role in digestive functions. Liver communicates something blocked and makes it flow to other organs. Pancreas absorbs nutrients and supplies them to organs and tissues throughout the body. It also promotes the circulation of body fluids and maintains water metabolism. Stimulating BL18 activates corticotropin-releasing hormone like neurons and modulates the expressions of corticotropin-releasing hormone and glucocorticoid receptor in the paraventricular nucleus of the hypothalamus. Electronic acupuncture on BL20 suppresses acid secretion and increases *β*-endorphin and somatostatin.^[35]^ In particular, BL22 features a traditional concept organ called triple-heater (San-jiao; Sam-cho; 三焦). San-jiao is divided into 3 parts. The 3 parts of the triple-heater are related to different functions, and the middle part named Zhong-jiao (Zung-cho, 中焦) is related to digestive functions. Therefore, we selected these 3 acupoints to analyze the digestive function of the back-shu points. According to these explanatory analyses, we presented an accurate method of inserting an acupuncture needle into the BL18 or BL20 for treating GI tract disease by inserting the needle into the lower part of the 12th rib from the same position as the vertical insertion into the upper part of the transverse process of 1st thoracic bone to the outer side.

In this paper, we systemically reviewed those 3 back-shu points related anatomical structures of these 3 back-shu points to analyze the relevance between physiological response and physical structure. In addition, by listing each study’s needling method, we analyzed the appropriate depth and angle of the needle to induce a therapeutic response. This research will help provide instructions on applying the needle to stimulate the related anatomical structure, inducing the therapeutic response.

Our study has several limitations. First, the quality of the included trials was not very high. Second, the sample size was not sufficiently large. Third, insufficient studies used medical devices to measure depth and angles accurately. Further follow-up clinical research is needed to develop accurate acupuncture needling guidelines.

However, this is the first systematic review to focus on the needling methods of BL18, BL20, and BL22. Although the inclusion of needle penetration depth and angle is still controversial, our study should help standardize acupuncture treatment strategies for GI disease. We also expect our results to be adopted by researchers who plan to conduct clinical trials using these acupoints as a set of standard needling anatomical targets or methods for accurate and less biased trials.

## Author contributions

Cho Y, Han Y, and Ki, Y: Methodology and data curation.

Han S and Oh K: Investigation.

Chae H: Writing – Review & editing.

Chu H: Writing– Original draft.

Ryu M: Supervision and project administration.

All authors approved the final version of the manuscript.

## Supplementary Material


