# Supplementary material for: Anatomical structures and needling method of the back-shu points BL18, BL20, and BL22 related to gastrointestinal organs: A PRISMA-compliant systematic review of acupoints and exploratory mechanism analysis
Source: Medicine (Baltimore). 2022 Oct 28;101(43):e29878. doi: 10.1097/MD.0000000000029878 (PMC9622668; doi:10.1097/MD.0000000000029878)
Supplement: Supplementary file 1 [file medi-101-e29878-s001.pdf]

Supplement 1. Characteristics of the included studies.

	First author (Year), Country	Language	Acupoint	Needling Depth	Needling angle	Needling Target or Anatomical Structures
1[38]	Jie S (2015), China	Chinese	BL 18	0.8寸	90°	muscle
2[39]	Jie S (2011), China	Chinese	BL 18	0.5~0.8寸	45°	muscle
3[41]	Gao X (2014), China	Chinese	BL 18<br>BL 20	0.5~0.8寸	45°	Targeting Spinal bone (向脊椎)
4[41]	Gao X (2014), China	Chinese	BL 18<br>BL 20	-	90°	muscle
5[42]	高希言(2011), China	Chinese	BL 18<br>BL 20	0.5~0.8寸	90°	muscle
6[43]	Qu (1996) China	Chinese	BL 20	3寸	-	muscle
7[44]	Qi (2017) China	Chinese	BL 18<br>BL 20	15~20mm<br>10~15mm	30~60°<br>90°	muscle
8[45]	Li (2018), China	Chinese	BL 18	0.8寸	90°	muscle
9[46]	Li (1999), China	Chinese	BL 18	1寸	Oblique insertion (斜刺)	Targeting Spinal bone (向脊椎)
10[47]	Li (2015), China	Chinese	BL 18	0.5~0.8寸	Oblique insertion (斜刺)	Targeting Spinal bone (向脊椎)
11[48]	Li (2017), China	Chinese	BL 20	-	45°	muscle
12[11]	Li (1995), China	Chinese	BL 18<br>BL 20<br>BL 22	-	-	BL 18: trapezius muscle, Middle accessory neurovascular bundle<br>BL 18 ,BL 20 ,BL 22: latissimus dorsi, latissimus dorsi Canal nerves (axillary arteries and veins)
13[49]	Li (2009), China	Chinese	BL 18<br>BL 20	1寸	Oblique insertion (斜刺)	Targeting Spinal bone (向脊椎)
14[50]	Li (2014), China	Chinese	BL 18<br>BL 20<br>BL 22	0.5~0.8寸	-	muscle
15[51]	Ma (2013), China	Chinese	BL 20	0.5~0.8寸	Oblique insertion (斜刺)	Targeting Spinal bone (向脊椎)
16[52]	Ma (2006), China	Chinese	BL 18<br>BL 20	1~1.5寸	Oblique insertion (斜刺)	Targeting Spinal bone (向脊椎)
17[53]	Mo (2020), China	Chinese	BL 18<br>BL 20	-	45°	muscle
18[54]	Wan (2019), China	Chinese	BL 18<br>BL 20	0.5~0.8寸	Oblique insertion (斜刺)	muscle

19[55]	Mou (2011), China	Chinese	BL 18<br>BL 20	0.5~0.8寸	45°	muscle
20[56]	Wu (2004), China	Chinese	BL 18	-	Oblique insertion (斜刺)	muscle
21[57]	Fang (2019), China	Chinese	BL 18<br>BL 20<br>BL 22	1.2~1.3寸	Oblique insertion (斜刺)	muscle
22[58]	Fu (2013), China	Chinese	BL 18<br>BL 20	0.5~0.8寸<br>0.5~1寸	Oblique insertion (斜刺)	muscle
23[59]	Fu (2020), China	Chinese	BL 18	0.2~0.5cm	-	Subcutaneous (局部出血)
24[60]	Ding (2011), China	Chinese	WBS	25-40mm	90°	muscle
25[61]	Zheng (2014), China	Chinese	BL18<br>BL20<br>BL22	1.5寸	-	muscle
26[62]	Zhao (1995), China	Chinese	BL22	1-2寸	Oblique insertion (斜刺)	Targeting Spinal bone (向脊椎)
27[63]	Zhao (2017), China	Chinese	BL18<br>BL20	1-2寸	75°	Targeting Spinal bone (向脊椎)
28[12]	Zhao (2010), China	Chinese	BL18	1寸 (Coronary artery disease)	Oblique insertion (斜刺)	Targeting Spinal bone (向脊椎)
			BL20	1.5-2寸 (Diabetes)		
			BL18	0.5-0.8寸 (Hypertension)		
			BL18,<br>BL20	0.5-0.8寸 (Constipation)		
29[64]	Zuo (2017), China	Chinese	BL18<br>BL20	1.5-2.5寸	75°	muscle
30[65]	Zhou (2017), China	Chinese	BL18<br>BL20	1寸	90°	muscle
31[66]	Zhu (2020), China	Chinese	BL20	0.5-0.8寸	Oblique insertion (斜刺)	Targeting Spinal bone (向脊椎)
32[67]	Zhou (2009), China	Chinese	BL18<br>BL20	0.8-1cm	Oblique insertion (斜刺)	Targeting Spinal bone (向脊椎)

33[68]	Zhu (2017), China	Chinese	BL19<br>BL20<br>BL22	0.8-1寸	Oblique insertion (斜刺)	muscle
34[69]	Zhou (2014), China	Chinese	BL18<br>BL20<br>BL22	0.8寸	45°	Targeting Spinal bone (向脊椎)
35[70]	ZENG (2019), China	Chinese	BL18<br>BL20	1.5-2.5寸	-	Targeting Spinal bone (向脊椎)
36[71]	Chen (2018)., China	Chinese	BL18<br>BL20	0.5-1寸	Oblique insertion (斜刺)	Targeting Spinal bone (向脊椎)
37[72]	Peng (2014), China	Chinese	BL20	0.8-1寸	90°	muscle
38[73]	Peng (2005), China	Chinese	WBS	15mm~30mm	Oblique insertion (斜刺)	Muscle, Targeting Spinal bone (向脊椎)
39[13]	Feng (2018), China	Chinese	WBS	Obese : 0.5 寸<br>Thin : 1 寸	90°	Muscle
40[74]	Feng (2020), China	Chinese	WBS	15~20mm	45°	Muscle
41[75]	Bi (2014), China.	Chinese	BL18	Oblique insertion (斜刺): 1寸<br>Vertical insertion: 0.5 寸	45°	Muscle
42[76]	He (1984), China	Chinese	BL18<br>BL20	1~1.5寸	-	muscle
43[16]	He (2003), China	Chinese	BL18<br>BL20<br>BL22	1.5寸	60°	muscle
44[77]	Xia (2006), China	Chinese	BL18<br>BL20<br>BL22	0.5~0.8寸	45°	muscle
45[78]	Huang F (2008), China	Chinese	BL18<br>BL20	20~25mm	Oblique insertion (斜刺)	Muscle, Targeting Spinal bone (向脊椎)

46[79]	Chen (2018), China	English	WBS	15~30mm	Oblique insertion (斜刺)	Muscle, Targeting Spinal bone (向脊椎)
47[80]	Qiu (2011), China	Chinese	WBS	10~15mm	45°	Muscle, Targeting Spinal bone (向脊椎)
48[81]	Chen (2008), China	Chinese	BS	0.5~ 0.8寸	45° or 90°	Muscle, Targeting Spinal bone (向脊椎)
49[82]	Chen (2014), China	Chinese	BL18<br>BL20	15~20mm	Oblique insertion (斜刺)	Muscle, Targeting Spinal bone (向脊椎)
50[83]	Xiao (2010), China	Chinese	BL20<br>BL22	1.0~1.2촌	90°	Muscle
51[14]	Cong (2014), China	Chinese	BL18	-	90° or<br>Oblique insertion (斜刺)	Muscle
52[84]	She (2009), China	Chinese	BS	20~40mm	-	Targeting Spinal bone (向脊椎)
53[85]	She (2009), China	Chinese	BL22	45~60mm	Oblique insertion (斜刺)	Targeting Spinal bone (向脊椎)
54[86]	Chang (2021), China	Chinese	BL18<br>BL20	-	45	muscle
55[87]	Chang (2019), China	Chinese	BS	0.5~0.8寸	Oblique insertion (斜刺)	muscle
56[88]	Xu (2013). China	Chinese	BL18<br>BL20	0.5~1寸	90	Targeting Spinal bone (向脊椎)
57[89]	Xu (2006), China	Chinese	BL18<br>BL20	1寸	Oblique insertion (斜刺)	Targeting Spinal bone (向脊椎)
58[15]	SHI (2017), China	Chinese	BL18<br>BL20	9~16mm	90	muscle
59[90]	Nie (2001), China	Chinese	BS	-	30	muscle
60[91]	Shao (2016), China	Chinese	BL18<br>BL20	13~20mm	90	Targeting Spinal bone (向脊椎)
61[92]	Xiao (2015), China	Chinese	BL20	25mm	90	muscle
62[93]	Su (2019), China	Chinese	BL20	0.5~0.8寸	45	Targeting Spinal bone (向脊椎)
63[94]	Song (2019), China	Chinese	BL18<br>BL20	0.5~0.8 Inch	45	muscle
64[95]	Yue (2008), China	Chinese	BL18<br>BL20	-	-	muscle
65[96]	Yang (2016), China	Chinese	JP, BS	1寸	45	Targeting Spinal bone (向脊椎)

66[97]	Yang (2014), China	Chinese	JP, BS	1.5寸	90	muscle
67[98]	Yang (2012), China	Chinese	BL18<br>BL20<br>BL22	-	90	muscle
68[99]	Wu (2020), China	Chinese	BL20	0.5~0.8寸	90	muscle
69[100]	Wu (2009), China	Chinese	JP, BS	-	30~45	Targeting Spinal bone (向脊椎)
70[101]	Wu (2015), China	Chinese	BL18<br>BL20	-	30	muscle
71[102]	Wu (2019), China	Chinese	BL20	10mm	45	muscle
72[103]	Wang (2012), China	Chinese	BS	0.5-0.8 Inch	45	Targeting Spinal bone (向脊椎)
73[104]	Wang (2019), China	Chinese	BL18	-	-	muscle
74[105]	Cui (2014), China	Chinese	BS	-	-	muscle
75[106]	Wang (2010), China	Chinese	BL18	1 Inch	-	muscle
76[107]	Wang (2020), China	Chinese	BS	0.5-1 Inch	-	muslce
77[108]	Wang (2014), China	Chinese	BL18<br>BL20	0.5-0.8	-	muscle
78[109]	Wang (2009), China	Chinese	BL18<br>BL20	1寸	-	muscle
79[110]	Wang (2019), China	Chinese	BL18<br>BL20	20-25mm	45	muscle
80[111]	Wei (2019), China	Chinese	BL18<br>BL20<br>BL22	BL18, BL20 0.5~0.8寸<br>BL22: 0.5~1寸	BL18, 20 : -<br>BL22 : 90	muscle
81[112]	Yin (2013), China	Chinese	BL18<br>BL20	0.8~1分	90	muscle
82[113]	Ren (2011), China	Chinese	BS	0.5~0.8 Inch	-	muscle
83[114]	Zhang (2012), China	Chinese	BL18	0.5~0.8 Inch	-	muscle
84[115]	Jiang (2021), China	Chinese	BL18<br>BL20	40~55mm	90	muscle
85[116]	Zhang, (2019), China	Chinese	BL18<br>BL20	13mm	-	muscle
86[117]	Zhang (2009), China	Chinese	BS	-	-	muscle

87[118]	Zhang (2007), China	Chinese	BL18<br>BL20	25mm	-	muscle
88[119]	Zhang (2010), China	Chinese	BL18<br>BL20	-	-	muscle
89[120]	Zhang (2011), China	Chinese	BL20	15mm, 25~30mm	-	muscle
90[121]	Zhang (2010), China	Chinese	BL18<br>BL20	0.8~1.0cm	-	Targeting Spinal bone (向脊椎)
91[122]	Zhang (2011), China	Chinese	BL18<br>BL20	0.8~1.0cm	-	Targeting Spinal bone (向脊椎)
92[123]	Jiang (2014), China	Chinese	BL18<br>BL20	15mm(BL18),<br>20mm(BL20)	-	muscle
93[124]	Zhang (2018), China	Chinese	BL18<br>BL20	1.5~2.0 Inch	45	Targeting Spinal bone (向脊椎)
94[125]	Zhang (2009), China	Chinese	BL18<br>BL20	1~2 Inch	30	muscle
95[126]	Tian (1994), China	Chinese	BS	-	90	muscle
96[127]	Qian (1991), China	Chinese	BL18	0.5寸	-	muscle
97[128]	Gong(2019), China	English	BL18	Intradermal needling	-	Muscle
98[129]	Takayama, S(2011), Japan	English	BL18	20mm	-	muscle
99[130]	Tang (2011), China	Chinese	BL 18<br>BL 20	0.5~1寸	90°	muscle
100[131]	DAI (2020), China	Chinese	BL 18<br>BL 20	25~30mm	60°	Targeting Spinal bone (向脊椎)
101[17]	Dong (2011), China	Chinese	BL 18<br>BL 20	-	90°	musculature lesion,<br>side of the tendon
102[132]	Lan (2012), China	Chinese	BL 18<br>BL 20	1寸	90°	muscle
103[133]	LU (2015), China	Chinese	BL 18<br>BL 20<br>BL 22	0.5~0.8寸	Oblique insertion (斜刺) or 90°	muscle

104[134]	Liu (2017), China	Chinese	BL 18<br>BL 20	0.5~0.8寸	Oblique insertion (斜刺)	parallel to the spinal bone
105[135]	Liu (2015), China	Chinese	BL 18<br>BL 20<br>BL 22	1.5~2.5cm	45°	Targeting Spinal bone (向脊椎)
106[136]	Liu (2013), China	Chinese	BL 18<br>BL 20	0.5~1寸	Oblique insertion (斜刺)	muscle
107[137]	Liu (2009), China	Chinese	BL 18<br>BL 20<br>BL 22	0.5~0.8寸	Oblique insertion (斜刺)	muscle
108[138]	Liu (2020), China	Chinese	BL 20	25mm	90°	muscle
109[139]	Liu (2013), China	Chinese	BL 18<br>BL 20	20mm	-	muscle
110[140]	Liu,(2007), China	Chinese	BL 18<br>BL 20<br>BL 22	0.5~1寸	45°	Subcutaneous (皮下)
111[141]	Liu (2021), China	Chinese	BL 18	12mm	30°	Targeting Spinal bone (向脊椎)
112[142]	Li (2014), China	Chinese	BL 18<br>BL 20	20~25mm	45°	Targeting Spinal bone (向脊椎)
113[143]	Li (2009), China	Chinese	BL 18<br>BL 20	0.8~1.2寸	90°	muscle
114[144]	Li (2011), China	Chinese	BL 18<br>BL 20	0.8~1.2寸	90°	muscle
115[18]	Li (2011), China	Chinese	BL 18			Cutaneous branches of posterior ramus of 9th, 10th thoracic nerve and related artery and vein`s soft tissue
